# Widespread venous thrombosis: Unveiling a complex case of Behçet’s disease with a literature perspective

**DOI:** 10.1515/med-2025-1199

**Published:** 2025-08-06

**Authors:** BinHan Liu, Chunyu Tan

**Affiliations:** Department of Rheumatology and Immunology, West China Hospital, Sichuan University, Chengdu, 610041, Sichuan, China

**Keywords:** Behçet’s disease, multisystem symptoms, pulmonary artery embolism, thrombosis, vasculitis, clinical improvement

## Abstract

**Background:**

Behçet’s disease, first described by Hippocrates and later recognized in modern medicine after Hulusi Behçet’s description in 1937, is a complex vasculitis often referred to as the “Silk Road” disease. It is more prevalent in the Middle East and Mediterranean regions, primarily affecting males aged 25–35.

**Case presentation:**

This study first describes the clinical presentation, timeline, diagnostic evaluation, and therapeutic interventions of one patient. Subsequently, the challenges in diagnosing the disease and selecting treatment strategies are discussed in detail. Finally, the clinical outcomes and treatment effects during the follow-up period are reported.

**Conclusion:**

This case underscores the necessity of comprehensive diagnosis and integrated treatment for diseases with complex multisystem symptoms. Multidisciplinary collaboration plays a crucial role in the management of such conditions.

## 1 Introduction

Behçet’s disease (BD), a multifaceted vasculitis, has been recorded since Hippocrates in ancient Greece [1,2,3]. However, it was not until 1937 that Turkish physician Hulusi Behçet clearly defined its contours in modern medicine [4]. Known as the “Silk Road” disease, the name of BD reflects its geographic distribution along the ancient trade route from the Middle East to the Mediterranean [5,6,7]. The clinical manifestations of this syndrome are diverse, including recurrent oral ulcers, genital ulcers, skin lesions, and ocular symptoms [8,9,10]. The complexity of the disease is underscored by its effects on different blood vessels, leading to arterial or venous complications, particularly venous thrombosis, which has been a significant focus in clinical research [11,12,13].

Previous studies have focused on its etiology, proposing a hypothesis of combined genetic predisposition and environmental triggers [14]. Some of these hypotheses suggest that autoimmune reactions might occur in genetically susceptible individuals when exposed to specific environmental factors, possibly infectious agents [15,16]. However, the exact pathogenesis of BD remains elusive, adding to the complexity of its diagnosis and treatment [17,18,19].

The disease may involve multiple systems, such as the central nervous system, vascular system, skin, and eyes; close cooperation among various specialties is essential to ensure that patients receive comprehensive and effective treatment [20,21]. This interdisciplinary approach enhances diagnostic accuracy and allows for the development of tailored treatment plans that best suit the patient’s needs [22].

This study aims to conduct a detailed case analysis of a patient with complex multisystem symptoms of BD, exploring the challenges and opportunities in diagnosing, treating, and managing the disease. Through the presentation of this case, we focus on the complexity of disease management and the importance of multidisciplinary team collaboration in instances where extensive venous thrombosis involves multiple organs. This research aims to deepen the understanding of BD by examining an atypical case, facilitating improved diagnosis and management, and ultimately leading to more effective treatment options for patients.

## 2 Case presentation

A 30-year-old male presented with symptoms including cough, occasional chest tightness, fatigue, fever, and shortness of breath, prompting attention from the hospital. Physical examination revealed multiple symptoms such as icteric sclera, mild jaundice, twisted veins visible on the abdominal wall, an enlarged palpable liver, and mild pitting edema in both lower limbs. Subsequent preliminary examination results indicated elevated transaminase levels (alanine transaminase 52 IU/L and aspartate transaminase 62 IU/L), suggesting potential liver failure. Laboratory findings further supported the diagnosis of liver dysfunction, including increased total bilirubin (34.4 μmol/L) and direct bilirubin (21.4 μmol/L), along with decreased albumin levels (27.4 g/L). Additionally, coagulation function tests revealed abnormal prothrombin time (16.6 s) and international normalized ratio (1.57), indicating significant liver impairment. However, further investigations revealed a more complex situation. Additional evaluations showed thrombosis in multiple areas of his body.

**Figure 1:**
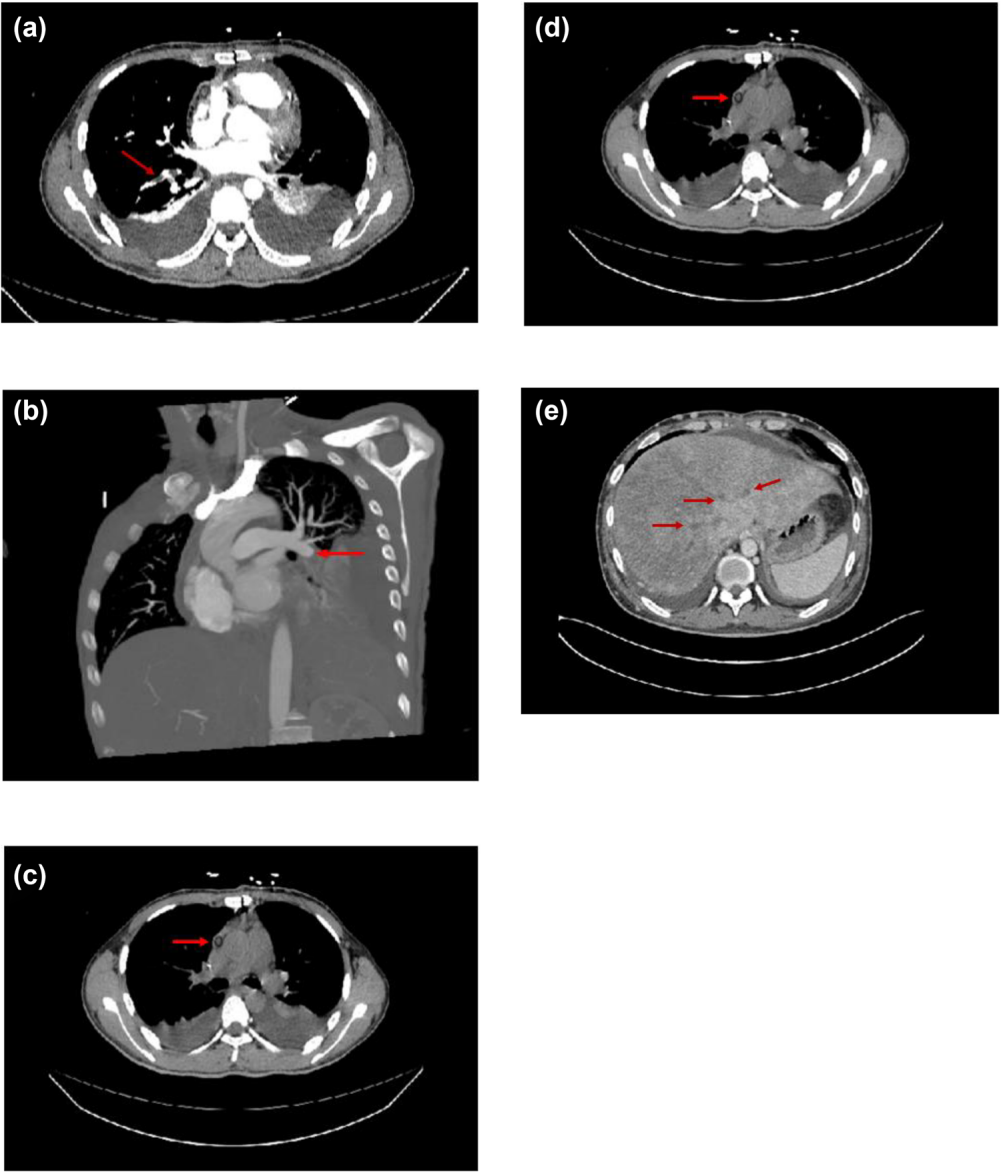
CTA imaging showing vascular filling defects. Note: (a) right pulmonary artery embolism, (b) left pulmonary artery thrombosis, (c) intracardiac thrombosis, (d) inferior vena cava thrombosis, and (e) thrombosis in the left, middle, and right hepatic veins.

Specifically, chest and abdominal computed tomography angiography (CTA) imaging indicated a significant filling defect in the lower lobe of the lung, consistent with pulmonary embolism, and filling defects caused by emboli were also observed in the right atrium, pulmonary artery left and middle hepatic veins, distal right hepatic vein, left common femoral vein, superficial femoral vein, and inferior vena cava (Figure 1). Despite attempts at interventional treatment, the procedure could not proceed due to bilateral femoral vein collapse, making thrombus removal surgery difficult. Therefore, the patient underwent a series of surgical procedures, including patent foramen ovale closure, right atrial thrombectomy, pericardial window drainage, thoracotomy, and sternotomy with internal fixation. Intraoperatively, a large thrombus (space-occupying lesion) was identified in the right atrium and successfully excised. Histopathological analysis revealed a mixed thrombus with necrosis and extensive neutrophil infiltration. For the thrombi in the pulmonary artery and hepatic veins, due to the high surgical risks, surgical thrombectomy was not performed. Instead, conservative treatment primarily involving low-molecular-weight heparin anticoagulation was adopted. Postoperatively, thrombus-related markers gradually improved; however, the patient developed recurrent fever, cough with sputum production, and the formation of pleural and abdominal effusions.

Wound healing was slow, and acne-like rashes appeared on his back. The surgical incision also showed mild oozing, and mild pitting edema was observed in both lower limbs. Meanwhile, sputum culture results revealed a multidrug-resistant strain of *Acinetobacter baumannii*. Additionally, immunological screening showed some abnormal results, including positive reactions for phosphatidylserine, annexin A5, and endothelial cell antibodies, although routine phospholipid antibody results were normal. Further diagnostic workup included serological tests, such as antinuclear antibody (ANA), extractable nuclear antigen, antineutrophil cytoplasmic antibody, and other autoimmune markers, all of which were negative; however, elevated levels of anti-protein C antibody, anti-phosphatidylethanolamine antibody, anti-annexin A5 antibody, and anti-endothelial cell antibody were detected. Interestingly, JAK2 V617F mutation testing and lupus anticoagulant tests were also negative. Thromboelastography results indicated generally normal coagulation function with minor abnormalities.

The diagnostic challenge initially lay in the suspicion of liver failure, which later required determining the underlying cause of multisystem thrombosis. After multidisciplinary discussions, multiple thrombi in the cardiac and hepatic vessels and femoral vein collapse suggested the possibility of vasculitis-associated occlusive vascular lesions. Additionally, the appearance of a rash on the patient’s back and positive laboratory findings for antiphospholipid and anti-endothelial cell antibodies, combined with imaging results, collectively pointed to a diagnosis of atypical Behcet’s Disease. Notably, the patient developed a postoperative multidrug-resistant *A. baumannii* infection, considered a postoperative complication. During the hospital stay, the patient received piperacillin–tazobactam and tigecycline for the treatment of *A. baumannii* pneumonia, low-molecular-weight heparin anticoagulation to address thrombosis, and human serum albumin to manage hypoalbuminemia and related complications. Methylprednisolone was administered as an anti-inflammatory therapy, with the dosage gradually increased from 20 to 80 mg/day. Eventually, after prolonged treatment, including steroids, antibiotics, anticoagulants, plasma, and albumin infusions, the patient showed a significant clinical improvement. Follow-up imaging revealed enhanced collateral circulation in the thoracic and abdominal walls, normalization of liver function, and only mildly elevated bilirubin levels. The patient was discharged 5 months later and received a comprehensive maintenance therapy regimen, including oral anticoagulant rivaroxaban (20 mg once daily), the immunomodulatory drug hydroxychloroquine (200 mg twice daily), and biweekly infusions of 10 g human albumin to correct hypoalbuminemia. Regular laboratory tests for liver function, inflammatory markers, and imaging studies were conducted to monitor thrombi and collateral circulation. During 12 months of follow-up, the patient demonstrated a significant improvement in symptoms, with no adverse events reported.

This case highlights the need to comprehensively consider various possibilities and employ integrated treatment approaches in complex multisystem thrombosis cases. Atypical BD emerged as a potential diagnosis. This case also underscores the importance of multidisciplinary collaboration in managing complex cases.


**Ethical approval:** This study was approved by the Biomedical Ethics Review Committee of West China Hospital, Sichuan University (No. 2023(1822)).
**Ethical statement:** This study was conducted strictly according to the ethical guidelines set forth by the Declaration of Helsinki and was approved by the local ethics committee, ensuring the ethical appropriateness and legality of the research design. All participants provided informed consent in writing after receiving a comprehensive explanation of the study’s objectives, potential risks, and their right to withdraw at any time. The highest protection was afforded to participants’ personal information and privacy, with all necessary measures taken to ensure data confidentiality and anonymity. The welfare of participants was prioritized throughout the study, with a system in place for the prompt reporting and management of any adverse events. The research team upheld principles of fairness and integrity at the highest standard, acknowledging participants’ contributions and the ethical review committee, which guaranteed the authenticity and reliability of the research findings while respecting the rights and welfare of all participants.

## 3 Discussion

BD is an autoimmune vasculitis that affects multiple organ systems, including the mucosa, blood vessels, skin, nervous system, gastrointestinal tract, eyes, and joints [23]. Vascular Behçet’s disease (VBD) is a manifestation of BD, with common symptoms including superficial venous thrombosis and deep vein thrombosis, affecting approximately 15–40% of patients with BD [24,25]. This study aims to explore atypical thrombosis and its treatment in BD through a complex case of VBD, particularly focusing on management strategies when multiple critical organs are involved. Cytokines such as interleukin-1, interleukin-6, interleukin-8, interleukin-17, and tumor necrosis factor-alpha are pivotal in BD pathophysiology, promoting a chronic inflammatory state leading to endothelial dysfunction and thrombotic events [26,27]. These findings are crucial for understanding BD’s pathological mechanisms and guiding therapeutic strategies.

In terms of diagnosis, diagnosing atypical BD is challenging because its symptoms do not fully meet the standard diagnostic criteria for BD and are often insidious. Typical symptoms of BD, such as oral ulcers, genital ulcers, ocular inflammation, and skin lesions, may not be present or may not fully align with diagnostic criteria; other symptoms may include abdominal pain, bloating, hematochezia, abdominal masses, diarrhea, and weight loss; arthritis, vasculitis, and neurological involvement. Clinically, diagnosis requires a comprehensive assessment of clinical manifestations, laboratory tests, and imaging results to avoid misdiagnosis [28]. The case underscores the importance of integrating clinical manifestations, imaging studies, and immunological markers to establish a definitive diagnosis.

The patient presented with extensive venous thrombosis, including pulmonary embolism, inferior vena cava thrombosis, and intracardiac thrombus, which raised suspicion of vasculitis – a hallmark feature of vascular-type BD. However, these manifestations are not exclusive to BD and may also be observed in conditions such as antiphospholipid syndrome (APS), systemic vasculitis, or infective endocarditis. Imaging studies revealed filling defects, collateral circulation formation, and the absence of atherosclerotic plaques in vessel walls, supporting a diagnosis of vasculitis while excluding embolic or atherosclerotic diseases. Negative results for lupus anticoagulant and ANA further ruled out APS and systemic lupus erythematosus (SLE). The absence of valvular vegetations and negative blood cultures also excluded infective endocarditis.

Immunological testing showed positivity for anti-phosphatidylserine and anti-Annexin V antibodies, indicating immune dysregulation and a pro-thrombotic state consistent with BD-related vasculitis. Literature reports that anti-phosphatidylserine antibodies are significantly elevated in patients with BD, which may have a potential role in diagnosing atypical BD [29]. Furthermore, anti-Annexin V antibodies, associated with reduced anticoagulant activity, could help assess disease activity in atypical BD [30]. While these markers lack specificity, they are critical in excluding other thrombotic disorders such as APS.

Given the high risk of vascular involvement, invasive procedures such as biopsies were avoided in this case. The diagnosis was entirely based on multidisciplinary imaging, immunological, and laboratory findings assessments. This case highlights the importance of multidisciplinary collaboration in managing atypical BD and underscores the need for further research to establish standardized diagnostic methods, particularly for patients primarily with vascular involvement.

Our case report highlights the importance of a multidisciplinary approach in diagnosing and managing complex and atypical BD cases. Especially in dealing with rare and severe thrombotic events, such as Budd-Chiari syndrome and intracardiac thrombosis, the collaboration of a multidisciplinary team is essential for achieving optimal clinical outcomes [31,32,33].

Our case analysis highlights the significant clinical improvement observed in patients following comprehensive treatment strategies, consistent with results reported in previous literature [34,35,36]. Notably, a 1-year follow-up in our study showed good recovery, further validating the effectiveness of a combined treatment approach that included anticoagulation, anti-inflammatory therapy with methylprednisolone, and supportive care.

**Figure 2:**
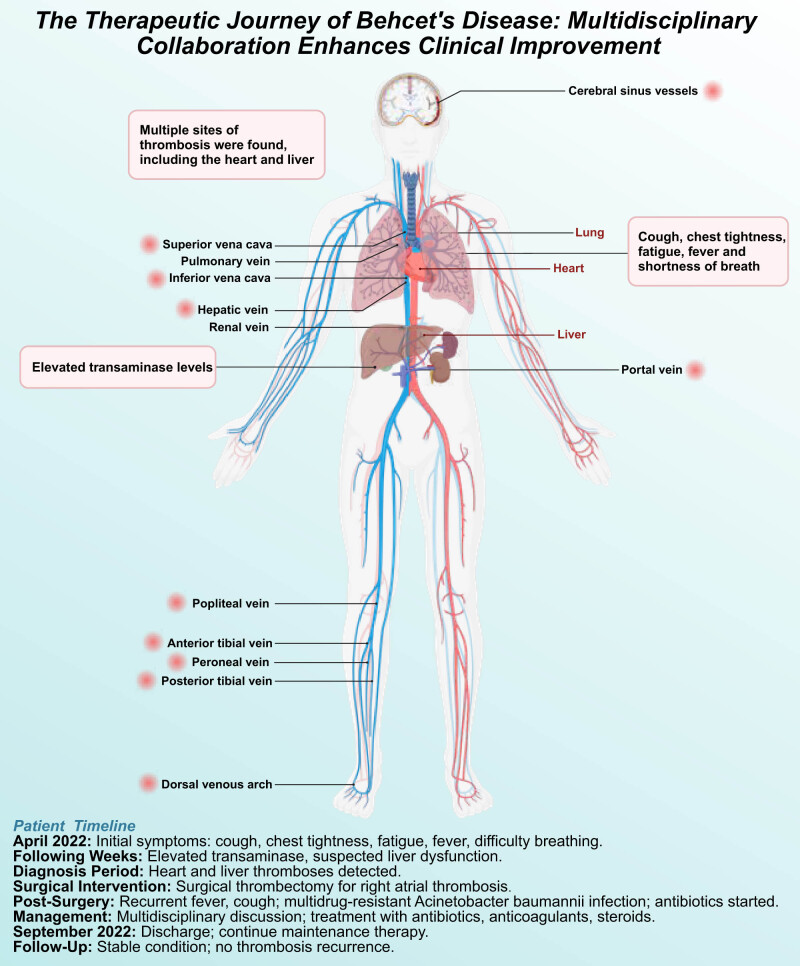
A graphical overview of widespread venous thrombosis and vascular BD.

This study provides significant clinical insights into treating BD, particularly in managing complex thrombotic events in VBD. Through a detailed analysis of a rare and complex VBD case, this study highlights the effectiveness of combined treatment strategies, including anticoagulation therapy and anti-inflammatory treatment with methylprednisolone, in achieving notable clinical improvement and preventing thrombus growth and recurrence. These findings emphasize the importance of tailoring treatment strategies to the individual needs of patients with BD and underscore the critical role of multidisciplinary team collaboration in optimizing clinical outcomes and improving the quality of life for patients with complex thrombotic complications (Figure 2).

Despite the valuable insights this study provides on BD treatment, there are certain limitations. First, this study is based on one case report, which may limit the generalizability of the results to all patients with BD. Additionally, due to a lack of long-term follow-up data, these treatment strategies’ long-term efficacy and potential side effects remain unclear. While this study emphasizes the advantages of immunosuppressive therapy, it does not fully compare the relative effectiveness and safety of all available treatment options, potentially limiting a comprehensive understanding of the optimal treatment strategies for BD. Finally, although the successful case of multidisciplinary collaboration in this study provides an effective management model, replicating this approach in different healthcare settings may be challenging, particularly in resource-limited environments.

Future research should focus on expanding sample sizes and conducting long-term follow-ups to more accurately evaluate BD treatment strategies, particularly the long-term effects and safety of immunosuppressive therapy and biological agents in preventing thrombus recurrence. Further randomized controlled trials comparing different treatment methods’ relative efficacy and safety are crucial for optimizing BD treatment guidelines. Additionally, in-depth research into the pathophysiology of BD, particularly the immunological basis of thrombosis, may reveal new therapeutic targets, guiding the development of novel treatment approaches. Exploring strategies for implementing multidisciplinary collaboration in different healthcare settings is also an important direction for future research, contributing to more effective management of complex diseases like BD. Finally, studies on patient education and self-management strategies should also be included in future work to improve patient treatment adherence and quality of life.
